# Long-Term Outcomes of Carbon Dioxide Insufflation in Thoracoscopic Esophagectomy After Neoadjuvant Chemotherapy for Esophageal Squamous Cell Carcinoma: A Retrospective Cohort Study

**DOI:** 10.7759/cureus.65053

**Published:** 2024-07-21

**Authors:** Koji Otsuka, Satoru Goto, Tomotake Ariyoshi, Takeshi Yamashita, Akira Saito, Masahiro Kohmoto, Rei Kato, Kentaro Motegi, Nobuyuki Yajima, Masahiko Murakami

**Affiliations:** 1 Esophageal Cancer Center, Showa University Hospital, Tokyo, JPN; 2 Department of Medicine, Division of Rheumatology, Showa University School of Medicine, Tokyo, JPN; 3 Department of Healthcare Epidemiology, School of Public Health in the Graduate School of Medicine, Kyoto University, Kyoto, JPN; 4 Center for Innovative Research for Communities and Clinical Excellence, Fukushima Medical University, Fukushima, JPN

**Keywords:** thoracoscopic esophagectomy, overall survival (os), long-term prognosis, esophageal cancer surgery, carbon dioxide insufflation

## Abstract

Background: Thoracoscopic esophagectomy (TE) with carbon dioxide (CO_2_)_ _insufflation is increasingly performed for esophageal cancer; however, there is limited evidence of the long-term outcomes of CO_2_ insufflation on postoperative survival.

Objectives: We investigated the long-term outcomes of TE with or without CO_2_ insufflation.

Methods: We enrolled 182 patients who underwent TE for esophageal cancer between January 2003 and October 2013 and categorized them into two groups: with and without CO_2_ insufflation. The primary endpoint was five-year overall survival (5y-OS). Secondary endpoints included long-term outcomes, such as five-year relapse-free survival (5y-RFS) and five-year cancer-specific survival (5y-CSS), and short-term outcomes, such as surgical and non-surgical complications and reoperation within 30 days.

Results: Follow-up until death or the five-year postoperative period was 98.9% (median follow-up duration was six years in survivors). After adjusting for age, sex, and yield pathologic tumor, node, and metastasis (TNM) stage, we found no significant differences in 5y-OS (HR 1.12, 95% CI 0.66-1.91), 5y-RFS (HR 1.12, 95% CI 0.67-1.83), or 5y-CSS rates (HR 1.00, 95% CI 0.57-1.75). For short-term outcomes, significant intergroup differences in operation time (p=0.02), blood loss (p<0.001), postoperative length of stay (p<0.001), and incidence of atelectasis (p=0.004) were observed. The results of the sensitivity analysis were similar to the main results.

Conclusions: In thoracoscopic procedures, CO_2_ insufflation significantly improved short-term outcomes, and it appears that the recurrence risk of esophageal cancer may not impact the long-term prognosis. While the influence of CO_2_ insufflation in thoracoscopic esophageal surgery remains unclear, our study suggests that the long-term prognosis is not compromised in other thoracic surgeries.

## Introduction

Esophageal cancer was the eighth most common cancer worldwide in 2018 [1]. Currently, there is an increasing incidence of esophageal cancer, and esophagectomy is an approved treatment. Cuschieri et al. reported the thoracoscopic resection of esophageal cancer in 1992 [2] and suggested the benefits of prone-position thoracoscopic esophagectomy (TE) in 1994 [3]. Thus, TE has been increasingly undertaken in recent years.

Intrathoracic carbon dioxide (CO_2_) insufflation during prone-position TE decreases lung compression, widens thoracic space visualization, and results in less oozing compared to TE without CO_2_ insufflation. However, the oncological effects of CO_2_ insufflation in thoracic procedures have not been clarified. CO_2_ insufflation in abdominal procedures is associated with tumor growth and is an independent risk factor for intravesical recurrence [4-6]. Conversely, other studies have suggested an association between CO_2_ insufflation and intrapneumoperitoneal apoptosis in human cancer cell lines [7-10]. While the short-term results of intrathoracic CO_2_ insufflation in esophageal cancer have been studied [11,12], the influence of intrathoracic CO_2_ insufflation on long-term survival remains unknown. Although rare, we have observed cases of distant metastasis following minimally invasive esophagectomy with CO_2_ insufflation, raising concerns about its potential role in such rare recurrences. Therefore, in this study, we investigated the long-term outcomes of intrathoracic CO_2_ insufflation during TE for esophageal cancer to understand the implications of its oncologic outcomes.

The primary objective was to ascertain the five-year overall survival (5y-OS) rate. The secondary objective was to determine the long-term prognosis, intraoperative factors, possibility of reoperation within 30 days, and 30-day mortality.

This article was previously posted to the Research Square preprint server on November 28, 2022.

## Materials and methods

Study design and patient selection

In this retrospective observational cohort study, we analyzed the surgical data of patients with squamous cell esophageal cancer treated with TE and neoadjuvant chemotherapy at the Showa University Hospital between January 2003 and October 2013. Based on the results of the Japan Clinical Oncology Group 9907 trial, neoadjuvant chemotherapy comprises two cycles of intravenous cisplatin (80 mg/m2) on days 1 and 22 and 5-fluorouracil (800 mg/m2) on days 1-5 and 22-26. Neoadjuvant chemotherapy with cisplatin plus 5-fluorouracil followed by radical surgery (within four weeks) is the standard treatment for resectable, clinical stage II/III esophageal cancer in Japan [13]. For clinical stage I cases, neoadjuvant chemotherapy is also considered for patients with suspected lymph node metastasis (cT1N1) or those requiring additional treatment following ESD due to the risk of postoperative recurrence.

We excluded patients with esophageal adenocarcinoma, small cell carcinoma, adenosquamous cell carcinoma, basaloid cell carcinoma, or other non-specified pathological types, as well as those with unresolved curative issues (e.g., residual tumors on pathological and macroscopic examination). Additionally, patients with early-stage esophageal cancer who did not require neoadjuvant chemotherapy, those with metastases to other organs (e.g., lung or liver), and tumor stage >T4b were excluded. Patients with serious cardiorespiratory diseases or other conditions that would preclude the safe conduct of surgery under general anesthesia were also excluded. Sequential sampling was performed. The study participants were assigned to two groups as follows: CO_2_ insufflation and non-CO_2_ insufflation.

Data collection

The baseline characteristics of the patients were analyzed and stratified based on intrathoracic CO_2_ insufflation. We obtained data on demographics, tumor location, clinical tumor, node, and metastasis (TNM) stage, yield pathologic TNM stage (yp TNM stage), neoadjuvant therapy, pleural adhesions, number of lymphadenectomy fields, reconstruction conduit, reconstruction route, anastomosis site, and conversion to thoracotomy, which were collected preoperatively or intraoperatively. Clinicopathological factors were classified according to the Union for International Cancer Control criteria, eighth edition [14], and complications were investigated using the Clavien-Dindo Classification (grade ≥2). Each clinical decision, including stage determination, was made by the attending physician. We accessed the database from July 1 to August 2, 2021.

Exposure

The exposure in this study was intrathoracic CO_2_ insufflation (pressure 8 mmHg), and the right lung was decompressed.

Outcome measures

The primary outcome was the 5y-OS rate. Secondary outcomes included other long-term outcomes, such as five-year relapse-free survival (5y-RFS) and five-year cancer-specific survival (5y-CSS), as well as short-term outcomes, such as thoracic operation time (minutes), thoracic blood loss (mL), blood transfusion (mL), time to extubation (days after surgery), postoperative length of stay (days), number of thoracic lymph nodes that were retrieved, total number of lymph nodes retrieved, number of positive lymph nodes, surgical complications (e.g., chylothorax, anastomotic leakage, postoperative bleeding, or recurrent laryngeal nerve paralysis), non-surgical complications (e.g., arrhythmia, atelectasis, pneumonia, or thrombosis), reoperation within 30 days, and 30-day mortality. To promote data independence, we only analyzed the initial event for each of the study outcomes. Relapse was determined by the attending physician through periodic imaging examinations during the postoperative follow-up period.

Anesthesia, position, and port arrangement

Surgery was performed under general anesthesia and in the left lateral decubitus position. Single-lung pulmonary ventilation using an 8-Fr spiral tube was performed, and a blocker was placed into the tube to block the right mainstem bronchus in both groups. The basic port arrangement included the insertion of 5-mm ports for the operator into the fifth and eighth intercostal spaces along the posterior axillary line, a 5-mm port for the thoracoscope in the eighth intercostal space in the midaxillary line, and 12-mm ports for the assistant in the slightly ventral third intercostal space and the fifth intercostal space on the anterior axillary line.

Thoracic procedure

Non-CO_2_ Insufflation Group

After inserting the small wound protection, we inserted the end-paddle (12 mm) R (Medtronic, Dublin, Ireland) through the 12 mm port in the fifth intercostal space along the anterior axillary line. The assistant surgeon compressed the right lung while using a suction device in the slightly ventral third intercostal space. The assistant’s role is crucial for ensuring optimal visualization during thoracic procedures.

CO_2_ Insufflation Group

**Figure 1:**
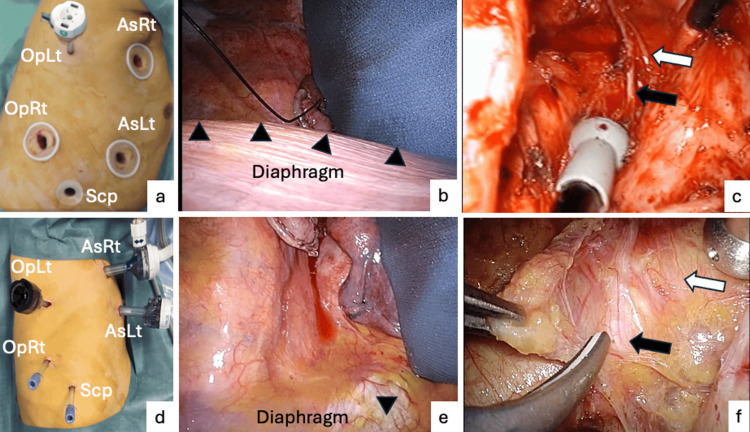
Port placement and thoracic view (a) Wound retractors are inserted in the no CO_2_ insufflation procedure. (b) The diaphragm (black triangle) disrupted the lower mediastinal view. (c) The left recurrent laryngeal nerve (black arrow) and cardiac blanch of the sympathetic nerve (white arrow) can be seen in the bloody view. (d) Three 5-mm and two 12-mm ports are inserted. AirSeal ports are inserted in a slightly ventral position in the third intercostal space at the anterior axillary line. (e) The picture is the same case with a. The diaphragm was pressed down after CO_2_ insufflation. (f) We can get a good view of the left recurrent laryngeal nerve (black arrow) and the cardiac branch of the sympathetic nerve (white arrow). Bleeding was decreased by CO_2_ insufflation pressure and helped with the view during the mediastinal lymph node dissection. OpLt: operator’s left-hand port, OpRt: operator’s right-hand port, AsRt: operator’s right-hand port, AsLt: operator’s left-hand port, Scp: scopist port

We used an AirSeal system (CONMED Corporation, Utica, NY, USA) that maintains a stable pneumothorax with continuous smoke evacuation and maintains a wide and clear intraoperative view despite using endoscopic suction. Following intrathoracic CO_2_ insufflation at a pressure of 8 mmHg, the right lung was decompressed, and lymph node dissection was performed around the right recurrent laryngeal nerve up to the level of the inferior thyroid artery. The esophagus was transected, and the lymph nodes around the left recurrent laryngeal nerve and tracheal bifurcation were dissected. Finally, the middle and inferior mediastinal lymph nodes, including the supradiaphragmatic and dorsal lymph nodes around the thoracic descending aorta, were dissected, and total mediastinal lymph node dissection was achieved (Figure 1).

Postoperative management

The tracheal tube was removed immediately after surgery. Patients were treated postoperatively in the intensive care unit for one day and then transferred to a high-care unit. Patients started walking and drinking water, resumed a liquid diet, returned to the general surgical ward, commenced a solid diet, and were discharged on postoperative days 1, 2, 3, 5, and ≥9, respectively. Recurrence was postoperatively assessed using CT examinations conducted every four to six months in the first two years and every six months thereafter [15].

Statistical analysis

Data are expressed as frequencies with percentages and medians (IQR) for categorical and continuous variables, respectively. Each factor was analyzed using the Mann-Whitney U or Fisher’s exact tests. Survival curves were prepared using the Kaplan-Meier method and compared using the log-rank test. Cox regression analysis was used to assess the association between CO_2_ insufflation and long-term prognosis (i.e., 5y-OS, 5y-RFS, and 5y-CSS), adjusted by age, sex, and yp TNM stage, to estimate the adjusted HR with a 95% CI. These confounding factors were chosen based on past literature and clinical importance [16-18]. Time zero was the day of the operation. The observation was concluded at death, the end of the study period, or if the patient was lost to follow-up. Patients were followed up for a maximum of five years. The proportional hazards assumption for the model was evaluated using the Schoenfeld test for assessing proportional hazards, which assesses the correlation between scaled residuals and time.

We conducted two sensitivity analyses to assess the robustness of the main results. First, we performed a Cox regression analysis adjusted for age, sex, and yp TNM stage using the cohort of participants who were registered during the transition from the non-CO_2_ insufflation procedure to the CO_2_ insufflation procedure (from December 1, 2010, to June 18, 2011) to factor in the changes in the treatment modalities over time during the decade evaluated by this study. Second, we performed propensity score matching to balance baseline covariates for the two comparator groups (the CO_2_ insufflation group and the non-CO_2_ insufflation group). We used 1:1 nearest-neighbor propensity score matching without replacement, using a caliper width that was 1/5 logit of the SD, with rigorous adjustment for significant differences in the baseline patient characteristics [19]. After checking the balance of covariates within the matched pairs, we used a structured iterative approach to refine the logistic regression model. We used the standardized difference to measure covariate balance and defined the imbalance as an absolute standardized difference higher than 10% [20]. The HR of the 5y-OS in the CO_2_ insufflation group compared to the non-CO_2_ insufflation group was estimated using a Cox proportional hazard regression model. Variables with an SD of 0.1 or higher were included as confounding factors in the multivariate analysis. A complete case analysis was performed after excluding patients with missing data in the exposure, outcome, or potential confounder variables. All statistical analyses were conducted using STATA 17.0 (Stata Corp LP, College Station, TX, USA). A two-sided p<0.05 was considered statistically significant.

Ethical considerations

This study was approved by the Institutional Review Board of Showa University School of Medicine (authorization number: 2256). All patient data were anonymized and de-identified prior to analysis, and the requirement for informed consent was waived.

## Results

**Figure 2:**
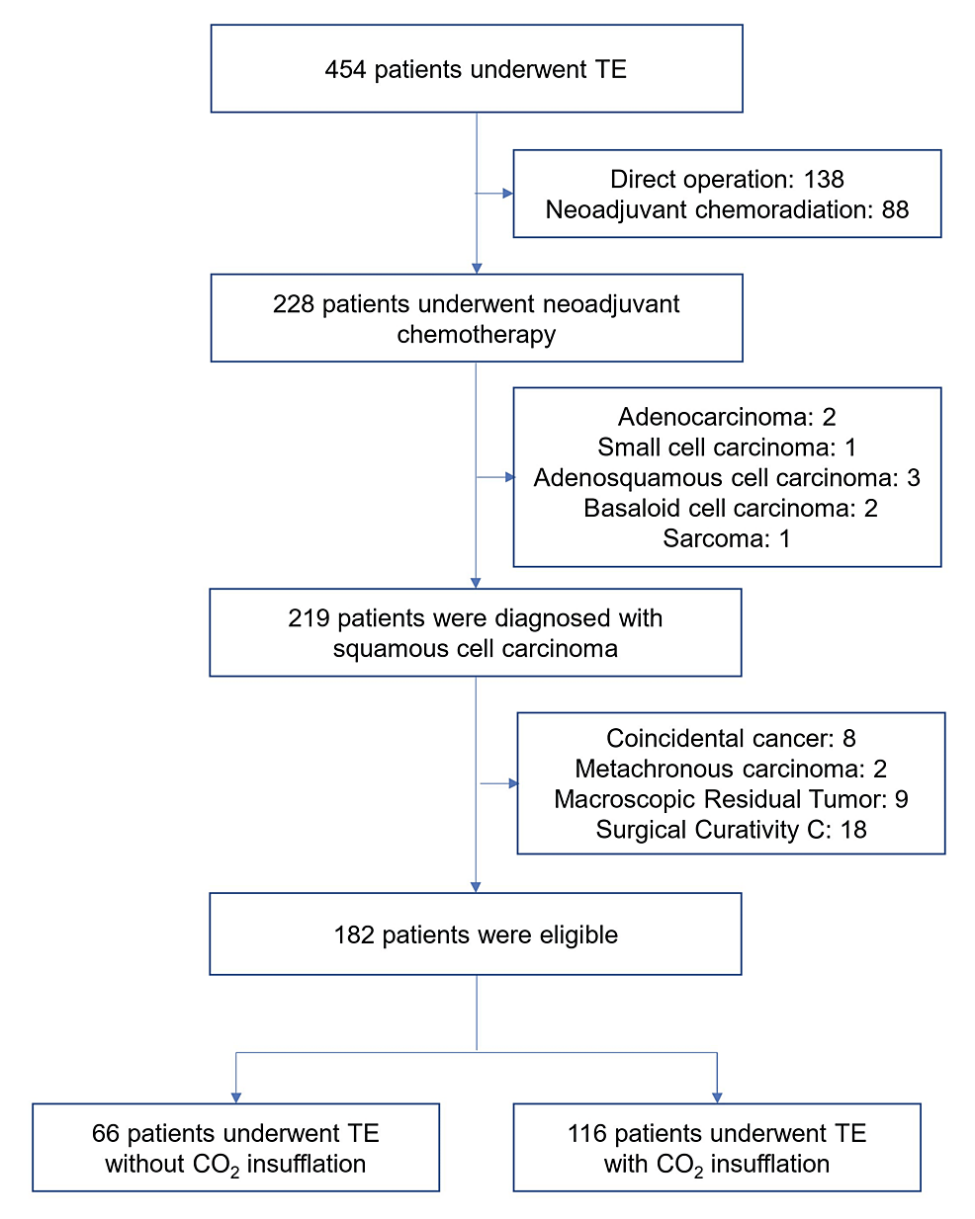
Patient selection flowchart Of the 454 patients who underwent TE for esophageal cancer, we excluded 265 patients. Finally, 182 patients were registered and assigned to the intrathoracic CO_2_ insufflation or non-CO_2_ insufflation groups. TE: thoracoscopic esophagectomy, CO_2_: carbon dioxide

As shown in Figure 2, we identified 454 patients who underwent TE for esophageal cancer during the study period. Of these, 265 were excluded for the following reasons: direct surgery without neoadjuvant chemotherapy (n=138), neoadjuvant chemoradiation (n=88), pathological findings (n=9), mixed cancer (n=10), and curative problems (pathological and macroscopic residual tumor; n=27). A total of 182 patients were enrolled in this study and assigned separately to the CO_2_ insufflation or non-CO_2_ insufflation groups. During the five-year postoperative period, two participants were lost to follow-up.

Baseline characteristics

The participant characteristics are shown in Table 1.

**Table 1:** Patient characteristics (n=182) IQR: interquartile range, SD: standard deviation, CO2: carbon dioxide, c TNM: clinical tumor, node, and metastasis, yp TNM: yield pathologic tumor, node, and metastasis

	Total	Missing data	CO_2_ insufflation group (n=116)	Non-CO_2_ insufflation group (n=66)	p
						Age, median (IQR)	66 (60-72)	0 (0.0%)	66 (60-72)	64 (59-70)	0.19
Male, n (%)	145 (79.7)	0 (0.0%)	90 (77.6)	55 (83.3)	0.44
Tumor location					0.51
Upper, n (%)	20 (10.9)	0 (0.0%)	11 (9.5)	9 (13.6)	
Middle, n (%)	111 (61.0)	0 (0.0%)	75 (64.7)	36 (54.5)	
Lower, n (%)	47 (25.8)	0 (0.0%)	27 (23.3)	20 (30.3)	
Abdominal, n (%)	4 (2.2)	0 (0.0%)	3 (3.4)	1 (1.5)	
c TNM stage					0.84
Stage I, n (%)	69 (37.9)	0 (0.0%)	42 (36.2)	27 (40.9)	
Stage II, n (%)	35 (19.2)	0 (0.0%)	24 (20.7)	11 (16.7)	
Stage III, n (%)	71 (39.0)	0 (0.0%)	46 (39.7)	25 (37.9)	
Stage IV, n (%)	7 (3.9)	0 (0.0%)	4 (3.4)	3 (4.5)	
yp TNM stage					0.02
Stage I, n (%)	74 (40.7)	0 (0.0%)	56 (48.3)	18 (27.3)	
Stage II, n (%)	41 (22.5)	0 (0.0%)	25 (21.6)	16 (24.2)	
Stage III, n (%)	48 (26.4)	0 (0.0%)	23 (19.8)	25 (37.9)	
Stage IV, n (%)	19 (10.4)	0 (0.0%)	12 (10.3)	7 (10.6)	
Pleural adhesions, n (%)	54 (29.8)	0 (0.0%)	38 (32.8)	16 (24.2)	0.31
Number of lymphadenectomy fields					0.58
Two, n (%)	111 (61.0)	0 (0.0%)	74 (63.8)	37 (56.1)	
Three, n (%)	71 (39.0)	0 (0.0%)	42 (36.2)	29 (43.9)	
Reconstruction conduit					0.08
Gastric tube, n (%)	177 (97.3)	0 (0.0%)	115 (99.1)	62 (93.9)	
Right colon, n (%)	3 (1.7)	0 (0.0%)	1 (0.9)	2 (3.0)	
Jejunum, n (%)	2 (1.1)	0 (0.0%)	0 (0)	2 (3.0)	
Reconstruction route					0.046
Retrosternal, n (%)	179 (98.4)	0 (0.0%)	116 (100)	63 (95.5)	
Posterior mediastinal, n (%)	3 (1.7)	0 (0.0%)	0 (0)	3 (4.5)	
Anastomosis site					0.13
Cervical, n (%)	180 (98.9)	0 (0.0%)	116 (100)	64 (97.0)	
Intrathoracic, n (%)	2 (1.1)	0 (0.0%)	0 (0)	2 (3.0)	
Conversion to thoracotomy, n (%)	0 (0)	0 (0.0%)	0 (0)	0 (0)	1

The median age of the patients was 66 (IQR 60-72) years, and 79.7% were male. Both study groups had similar patient characteristics at baseline. However, the CO_2_ insufflation group had an earlier yp TNM stage than the non-CO_2_ insufflation group. The postoperative follow-up until death or five years was 98.9% complete (180 of 182; reasons for a loss to follow-up are shown in Table 2), and the median follow-up duration was 6.0 (IQR 3.3-7.1) years.

**Table 2:** Reasons for ceasing patient follow-up CO_2_: carbon dioxide

	CO_2_ insufflation (n=116)	Non- CO_2_ insufflation (n=66)
	n (%)	n (%)
Death (all-cause)	45 (38.8)	30 (45.5)
Death (cancer-specific)	37 (31.9)	25 (37.9)
Followed for five years	70 (60.3)	35 (53.0)
Lost of follow-up	1 (0.9)	1 (1.5)

Five-year overall survival

**Figure 3:**
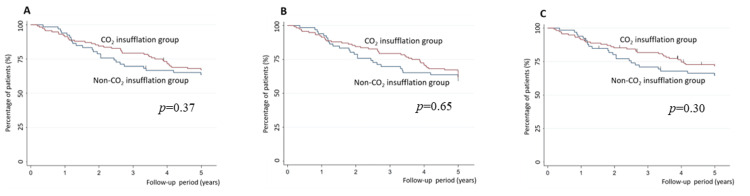
Kaplan–Meier estimates of long-term outcomes Kaplan-Meier estimates of long-term outcomes. (A) Kaplan-Meier curves for the 5y-OS with or without CO_2_ insufflation. There is no significant intergroup difference in the analysis using the log-rank test (p=0.37). (B) Kaplan-Meier curves for above 5y-RFS with or without CO_2_ insufflation show no significant differences between groups using the log-rank test (p=0.40). (C) Kaplan-Meier curves for 5y-CSS with or without CO_2_ insufflation show no significant intergroup difference using the log-rank test (p=0.30). CO_2_: carbon dioxide, 5y-OS: five-year overall survival, 5y-RFS: five-year relapse-free survival, 5y-CSS: five-year cancer-specific survival

Of the 182 patients, 62 (34.4%) died during the five-year postoperative period. Of the 62 deaths, 55 were due to esophageal cancer, and nine were due to other causes. Figure 3A shows the respective Kaplan-Meier curves for the 5y-OS in the groups with and without CO_2_ insufflation. No significant intergroup difference was detected using the log-rank test (p=0.37).

The Cox models showed that, after adjustment for age, sex, and yp TNM stage, the HR for CO_2_ insufflation was not statistically significant for the 5y-OS (HR 1.12, 95% CI 0.66-1.91) (Table 3).

**Table 3:** Association between CO2 insufflation and long-term survival Cox hazard models were constructed to estimate the HR, after adjustment for age and sex. Ref: reference, HR: hazard ratio, CI: confidence interval, CO_2_: carbon dioxide, 5y-OS: five-year overall survival, 5y-RFS: five-year relapse-free survival, 5y-CSS: five-year cancer-specific survival

	Non-CO_2_ insufflation group (n=66)	CO_2_ insufflation group (n=116)		
Long-term survival		Unadjusted HR	Adjusted HR	95% CI
5y-OS	Ref.	0.85	1.12	0.66–1.91
5y-RFS	Ref.	0.9	1.12	0.67–1.83
5y-CSS	Ref.	0.75	1	0.57–1.75

Other long-term outcomes

Among the 182 patients, 72 (39.6%) experienced cancer recurrence or all-cause death in the five-year postoperative period. Figure 3b shows the Kaplan-Meier curve for the 5y-RFS for the CO_2_ insufflation and non-CO_2_ insufflation groups, and no significant intergroup difference was detected using the log-rank test (p=0.65). In the Cox regression analysis adjusted for age, sex, and yp TNM stage, the 5y-RFS did not significantly differ between the two study groups (HR 1.12, 95% CI 0.67-1.83) (Table 3).

Of the 182 participants, 55 (31.0%) died because of esophageal cancer within five years after the TE. Figure 3c shows Kaplan-Meier curves for the 5y-CSS in the CO_2_ insufflation and non-CO_2_ insufflation groups. The log-rank test showed that the 5y-CSS did not differ significantly between the groups (p=0.30). Cox regression analysis adjusted for age, sex, and yp TNM stage showed no significant intergroup differences with regard to the 5y-CSS (HR 1.00, 95% CI 0.57-1.75) (Table 3).

Short-term outcomes

Table 4 shows the surgical outcomes and postoperative complications in the study groups. There were significant intergroup differences in the thoracic operation time (p=0.02), thoracic blood loss (p<0.001), postoperative length of hospital stay (p<0.001), and incidence of atelectasis (p=0.004).

**Table 4:** Short-term outcomes and postoperative complications (n=182) IQR: interquartile range, CO_2_: carbon dioxide

	Total	CO_2_ insufflation group (n=116)	Non-CO_2_ insufflation group (n=66)	p
					Thoracic operative time (minutes), median (IQR)	215 (180–265)	210 (170–260)	232.5 (200–280)	0.02
Thoracic blood loss (mL), median (IQR)	85 (50–150)	50 (32.5–100)	150 (100–220)	<0.001
Time to extubation (days after surgery), median (IQR)	0 (0–0)	0 (0–0)	0 (0–0)	0.92
Postoperative length of stay (days), median (IQR)	17 (15–21)	16 (13–19)	17 (16–24)	<0.001
Number of retrieved thoracic lymph nodes, median (IQR)	28 (20–36)	29 (23–36)	27.5 (23–34)	0.61
Number of retrieved total lymph nodes, median (IQR)	55 (44–75)	57 (45–74.5)	54 (43–76)	0.89
Number of positive lymph nodes, median (IQR)	1 (0–3)	0 (0–2)	0 (0–3)	0.02
Surgical complications				
Chylothorax, n (%)	3 (1.7)	2 (1.7)	1 (1.5)	1
Anastomotic leakage, n (%)	3 (1.7)	2 (1.7)	1 (1.5)	1
Postoperative bleeding, n (%)	5 (2.8)	3 (2.6)	2 (3.0)	1
Recurrent laryngeal nerve paralysis, n (%)	2 (1.1)	2 (1.7)	0 (0)	0.54
Non-surgical complications				
Arrhythmia, n (%)	3 (1.7)	2 (1.7)	1 (1.5)	1
Atelectasis, n (%)	15 (8.2)	4 (3.4)	11 (16.7)	0.004
Pneumonia, n (%)	12 (6.6)	5 (4.3)	7 (10.6)	0.12
Thrombosis, n (%)	2 (1.1)	0 (0)	2 (1.7)	0.13
Reoperation within 30 days, n (%)	7 (3.9)	4 (3.4)	3 (4.6)	0.71
Mortality within 30 days, n (%)	0 (0)	0 (0)	0 (0)	1

Sensitivity analysis

Sixty-four participants underwent TE in the transition period from non-CO_2_ insufflation to CO_2_ insufflation (from December 1, 2010, to June 18, 2011) at the study center. The Cox model analysis of these participants showed that after adjustment for age, sex, and yp TNM stage, the HR for intrathoracic CO_2_ insufflation was not statistically significant for the 5y-OS (HR 0.72, 95% CI 0.29-1.83).

In the assessment using propensity score matching, the covariate of the yp TNM stage was not balanced. Therefore, we conducted Cox regression analysis adjusting for the yp TNM stage, and the HR for intrathoracic CO_2_ insufflation was not signiﬁcantly associated with the 5y-OS (HR 0.74, 95% CI 0.38-1.43).

## Discussion

In this study, we investigated the long-term outcomes in patients who did or did not receive intrathoracic CO_2_ insufflation during TE. We did not observe significant intergroup differences in the 5y-OS, 5y-RFS, and 5y-CSS. However, there were significant differences in the thoracoscopic operation time, thoracic blood loss, postoperative length of hospital stay, and incidence of postoperative atelectasis.

The results suggest that CO_2_ insufflation is a safe assistive method when performing TE and does not influence long-term survival. Similar results to those of our research have been reported for CO_2_ insufflation in abdominal procedures [21-25]; however, several other studies have suggested that high-pressure pneumoperitoneum and prolonged pneumoperitoneum time were associated with tumor growth and intravesical recurrences [4-6]. There are several explanations for the results obtained in the present study. First, the lower incidence of postoperative atelectasis and pneumonia, which is associated with long-term outcomes [26], may have affected the long-term prognosis. In our short-term outcome assessment, there was a significantly reduced incidence of atelectasis in the CO_2 _insufflation group, although this was identified in univariate analysis. This reduction can be attributed to the continuous pressure of CO_2_ insufflation, which minimizes the need for forceful lung expansion to maintain adequate ventilation.

Second, the low CO_2_ pressure may have affected the long-term prognosis. A study on esophageal cancer suggested that CO_2_ insufflation promoted the viability, invasion, and metastasis of esophageal cancer cells, which was related to the insufflation pressure [27]. Specifically, high-pressure (12 mmHg) intrathoracic CO_2_ insufflation significantly increased cell viability, cell invasion, and metastasis of esophageal cancer cells, and moderate-pressure (8 mmHg) intrathoracic CO_2_ insufflation was safe and feasible [27]. In our institution, we performed TE with moderate-pressure (8 mmHg) CO_2_ insufflation; this is possibly why CO_2_ insufflation induced no significant differences in the RFS and OS. Third, several studies have suggested that CO_2_ insufflation is related to apoptosis in human cancer cell lines in the regions with pneumoperitoneum [7-10]. Moreover, CO_2_ insufflation may have positively affected the long-term prognosis, although the difference was not significant.

In our study, CO_2_ insufflation significantly reduced the thoracoscopic operation time. However, several studies indicated that CO_2_ insufflation did not improve the operation time [11,12]. There are several potential reasons for the lower thoracoscopic operation time observed in the present study. First, pressure was applied to widen the surgical field of view, thereby facilitating the performance of surgical techniques and an understanding of the microanatomical layer around the recurrent laryngeal nerve to prevent the complications described in our previous report [28]. Second, the methods of CO_2_ insufflation were different from those reported previously. The method of CO_2_ delivery influences whether the pressure can be maintained, which, in turn, affects the bleeding. Ninomiya et al. suggested that mediastinal deviation and the limitation of suction may be attributable to the limited effect of intrathoracic CO_2_ insufflation on shortening the operation time. We used an AirSeal system that maintained a stable pneumothorax with continuous smoke evacuation and maintained a wide and clear operation view without limitations. Therefore, the stable pressure may have helped secure the surgical field and allowed the appropriate procedure to be performed. Third, reduced blood loss would have shortened the operation time.

We observed significant improvements in thoracic blood loss using this procedure. Ninomiya et al. reported a similar result [11]. However, Mao et al. reported no improvement in thoracic blood loss [12]. There are several potential reasons for the lower thoracic blood loss in the present study. First, the CO_2_ insufflation pressure may affect small-vessel hemorrhage, thereby improving hemostasis during laparoscopic surgery [29]. Second, preventing respiratory acidosis may have reduced the bleeding tendency. Mao et al. suggested that CO_2_ insufflation affects coagulation by reducing the rate of blood clot formation and the intensity of blood clot agglutination caused by respiratory acidosis [12]. In our institution, we monitor end-tidal CO_2_ levels and adjust respiratory rates to prevent respiratory acidosis. Thus, there is a possibility that adaptive ventilation control prevented the impairment of coagulation in this study.

The use of AirSeal is known to contribute to hypothermia. While we did not monitor temperatures in this study, previous Japanese research compared the short-term outcomes between normal insufflation machines and AirSeal in thoracic procedures, finding significantly higher rates of hypothermia with AirSeal. However, we mitigated this effect by placing a whole-body blanket under the patient and heating it to 42 degrees Celsius after repositioning, resulting in normal temperature recovery by the end of surgery. Importantly, no significant differences were found in time to extubation or postoperative hospital stay length in our study, suggesting that hypothermia did not affect our present outcomes.

Strengths and limitations of the study

This study has several strengths. First, to our knowledge, this is the first clinical study to evaluate the role of CO_2_ insufflation during TE in terms of long-term outcomes. Second, only two patients were lost to follow-up, and the remaining 180 patients (98.9%) were followed up until either death or study completion. The two cases of loss of follow-up were due to relocation to an unspecified destination. Third, two sensitivity analyses were performed to ensure robust results.

This study has several limitations. First, this was a retrospective, single-center study. Further prospective, randomized, controlled studies are needed to understand the effect of intrathoracic CO_2_ insufflation. Second, the two groups (with or without CO_2_ insufflation) were not treated during the same study period. However, we performed an additional sensitivity analysis for participants who were registered during the transitional period and found no significant differences in long-term survival with or without CO_2_ insufflation. Third, the operation was not performed by the same surgeon. Therefore, this study did not consider the influence of the surgeon’s techniques. However, we had previously reported our results after standardizing the techniques, and three esophageal surgeons have performed this technique so far, reducing the possibility of inter-operator differences [30].

## Conclusions

In thoracoscopic procedures, CO_2_ insufflation significantly improved short-term outcomes, and it appears that the recurrence risk of esophageal cancer may not impact the long-term prognosis. While the influence of CO_2_ insufflation in thoracoscopic esophageal surgery remains unclear, our study suggests that the long-term prognosis is not compromised in other thoracic surgeries. Further prospective studies are needed to clarify the influence of intrathoracic CO_2_ insufflation.

## References

[1] Bray F, Ferlay J, Soerjomataram I, Siegel RL, Torre LA, Jemal A (2018). Global cancer statistics 2018: GLOBOCAN estimates of incidence and mortality worldwide for 36 cancers in 185 countries. CA Cancer J Clin.

[2] Cuschieri A, Shimi S, Banting S, Vander Velpen G (1993). Endoscopic ultrasonic dissection for thoracoscopic and laparoscopic surgery. Surg Endosc.

[3] Cuschieri A (1994). Thoracoscopic subtotal oesophagectomy. Endosc Surg Allied Technol.

[4] Shigeta K, Kikuchi E, Hagiwara M (2017). Prolonged pneumoperitoneum time is an independent risk factor for intravesical recurrence after laparoscopic radical nephroureterectomy in upper tract urothelial carcinoma. Surg Oncol.

[5] Gutt CN, Kim ZG, Hollander D, Bruttel T, Lorenz M (2001). CO2 environment influences the growth of cultured human cancer cells dependent on insufflation pressure. Surg Endosc.

[6] Krause P, Bobisch NS, Thelen P, Koehler K, Koenig S, Becker H, Leister I (2011). The plasminogen activator inhibitor system in colon cancer cell lines is influenced by the CO2 pneumoperitoneum. Int J Colorectal Dis.

[7] Ma JJ, Feng B, Zhang Y (2009). Higher CO2-insufflation pressure inhibits the expression of adhesion molecules and the invasion potential of colon cancer cells. World J Gastroenterol.

[8] Leng J, Lang J, Jiang Y, Liu D, Li H (2006). Impact of different pressures and exposure times of a simulated carbon dioxide pneumoperitoneum environment on proliferation and apoptosis of human ovarian cancer cell lines. Surg Endosc.

[9] Yang CK, Guan S, Ying MG (2014). Effects of CO₂ pneumoperitoneum on the expression of thymidine kinase 1 and Ki67 in colorectal carcinoma cells. Surg Endosc.

[10] Tan BJ (2006). Is carbon dioxide insufflation safe for laparoscopic surgery? A model to assess the effects of carbon dioxide on transitional-cell carcinoma growth, apoptosis, and necrosis. J Endourol.

[11] Ninomiya I, Okamoto K, Fushida S (2017). Efficacy of CO(2) insufflation during thoracoscopic esophagectomy in the left lateral position. Gen Thorac Cardiovasc Surg.

[12] Mao QX, Guo W, Huang BQ, Yan H (2016). Impact of artificial capnothorax on coagulation in patients during video-assisted thoracoscopic esophagectomy for squamous cell carcinoma. Surg Endosc.

[13] Ando N, Kato H, Igaki H (2012). A randomized trial comparing postoperative adjuvant chemotherapy with cisplatin and 5-fluorouracil versus preoperative chemotherapy for localized advanced squamous cell carcinoma of the thoracic esophagus (JCOG9907). Ann Surg Oncol.

[14] Rice TW, Ishwaran H, Ferguson MK, Blackstone EH, Goldstraw P (2017). Cancer of the esophagus and esophagogastric junction: an eighth edition staging primer. J Thorac Oncol.

[15] Kitagawa Y, Uno T, Oyama T (2019). Esophageal cancer practice guidelines 2017 edited by the Japan esophageal society: part 2. Esophagus.

[16] Xie SH, Santoni G, Mälberg K, Lagergren P, Lagergren J (2021). Prediction model of long-term survival after esophageal cancer surgery. Ann Surg.

[17] Han W, Deng W, Wang Q (2022). Applying post-neoadjuvant pathologic stage as prognostic tool in esophageal squamous cell carcinoma. Front Oncol.

[18] Hsu PK, Chen HS, Liu CC, Wu SC (2018). Application of the eighth AJCC TNM staging system in patients with esophageal squamous cell carcinoma. Ann Thorac Surg.

[19] Austin PC (2011). Optimal caliper widths for propensity-score matching when estimating differences in means and differences in proportions in observational studies. Pharm Stat.

[20] Austin PC (2007). Propensity-score matching in the cardiovascular surgery literature from 2004 to 2006: a systematic review and suggestions for improvement. J Thorac Cardiovasc Surg.

[21] Kim HH, Han SU, Kim MC (2019). Effect of laparoscopic distal gastrectomy vs open distal gastrectomy on long-term survival among patients with stage I gastric cancer: the Klass-01 randomized clinical trial. JAMA Oncol.

[22] Ng SS, Lee JF, Yiu RY (2014). Long-term oncologic outcomes of laparoscopic versus open surgery for rectal cancer: a pooled analysis of 3 randomized controlled trials. Ann Surg.

[23] Lewin JW, O'Rourke NA, Chiow AK, Bryant R, Martin I, Nathanson LK, Cavallucci DJ (2016). Long-term survival in laparoscopic vs open resection for colorectal liver metastases: inverse probability of treatment weighting using propensity scores. HPB (Oxford).

[24] Yoon YI, Kim KH, Cho HD (2020). Long-term perioperative outcomes of pure laparoscopic liver resection versus open liver resection for hepatocellular carcinoma: a retrospective study. Surg Endosc.

[25] Conrad C, Basso V, Passot G (2017). Comparable long-term oncologic outcomes of laparoscopic versus open pancreaticoduodenectomy for adenocarcinoma: a propensity score weighting analysis. Surg Endosc.

[26] Kataoka K, Takeuchi H, Mizusawa J (2017). Prognostic impact of postoperative morbidity after esophagectomy for esophageal cancer: exploratory analysis of Jcog9907. Ann Surg.

[27] Jiang T, Lin M, Zhan C, Zhao M, Yang X, Li M, Feng M (2019). High-pressure artificial pneumothorax promotes invasion and metastasis of oesophageal cancer cells. Interact Cardiovasc Thorac Surg.

[28] Otsuka K, Murakami M, Goto S (2020). Minimally invasive esophagectomy and radical lymph node dissection without recurrent laryngeal nerve paralysis. Surg Endosc.

[29] Teshima J, Miyata G, Kamei T (2015). Comparison of short-term outcomes between prone and lateral decubitus positions for thoracoscopic esophagectomy. Surg Endosc.

[30] Murakami M, Otsuka K, Goto S, Ariyoshi T, Yamashita T, Aoki T (2017). Thoracoscopic and hand assisted laparoscopic esophagectomy with radical lymph node dissection for esophageal squamous cell carcinoma in the left lateral decubitus position: a single center retrospective analysis of 654 patients. BMC Cancer.

